# Dietary diversity and its associated factors among rural labourers in South India

**DOI:** 10.3389/fnut.2025.1729962

**Published:** 2026-01-12

**Authors:** Settipalli Sravani, V. Lenin, Pratibha Joshi, Satyapriya, Mrinmoy Ray, Sitaram Bishnoi, Monika Wason, P. Punitha, K. Raghavendra Chowdary, M. Chenna Madhava, Venkata Naga Sindhuja Padigapati, Kotha Shravani, Sweety Mukherjee, Seema Kujur, Sakaray Vaishnavi, Rajat Kumar Nath

**Affiliations:** 1 Division of Agricultural Extension, ICAR-Indian Agricultural Research Institute, New Delhi, India; 2 Centre for Agricultural Technology Assessment and Transfer (CATAT), ICAR-Indian Agricultural Research Institute, New Delhi, India; 3 Agricultural Knowledge Management Unit, ICAR-Indian Agricultural Research Institute, New Delhi, India; 4 Krishi Vigyan Kendra, Banavasi, Kurnool District, Andhra Pradesh, India; 5 Department of Extension Education, Banaras Hindu University, Varanasi, India; 6 Division of Agricultural Economics, ICAR-Indian Agricultural Research Institute, New Delhi, India; 7 Agricultural Extension, Krishi Vigyan Kendra, East Sikkim, Sikkim, India

**Keywords:** Anthropometric indicators, Agricultural labourers, dietary diversity, nutritional status, public distribution system, rural labourers, South India, undernutrition

## Abstract

**Background:**

Malnutrition continues to be a major global health challenge affecting millions of vulnerable populations across countries. Despite their critical contribution to agricultural productivity, limited evidence exists regarding the dietary diversity and nutritional status of rural labourers in South India. Therefore, the present study aimed to assess dietary diversity and its associated factors among rural labourers in South India.

**Methods:**

A community-based cross-sectional study was conducted among 320 rural labourers (men aged 15–54 years and women aged 15–49 years, excluding pregnant and lactating women) and those who were actively engaged in farming and household activities. Respondents were selected using a multistage random sampling method. Data were collected through a structured interview schedule, and the collected data were entered into Excel and analyzed using R Studio (v4.2.2). The dietary diversity score was computed based on the 24-h recall method. Nutritional status was analyzed using the body mass index (BMI), mid-upper arm circumference (MUAC), and calf circumference. Pearson’s chi-squared test was used to determine the association between dietary diversity and nutritional status at a significance level of *p* < 0.05.

**Results:**

The findings of this study revealed that the dietary diversity among rural labourers was limited, with heavy reliance on locally available staple foods. In Andhra Pradesh, the majority of men (63.75%) and women (78.75%) had medium dietary diversity; however, men had relatively better diversity, with 21.25% attaining high dietary diversity compared to only 1.25% of women. Comparatively, in Telangana, the majority of men (68.75%) and women (52.50%) were also in the medium dietary diversity category. Although a larger portion of women (30.00%) were in the lowest category, 17.50% of women had high dietary diversity. This reduced the gender gap that existed in Andhra Pradesh. A significant association was observed between dietary diversity and nutritional status of rural labourers (*p* < 0.05).

**Conclusion:**

The study highlighted the low dietary diversity and the existence of undernutrition among rural labourers in South India. This emphasises the need for nutrition education, the promotion of household and community nutrition gardens, and greater awareness of balanced diets, all of which could help improve dietary intake. Furthermore, implementing nutrition and health awareness programmes together with anthropometric indices can aid policymakers in assessing the effectiveness of the Public Distribution System. These efforts can be the basis for the development of targeted interventions that will not only change food consumption patterns but also improve the nutritional status of rural labourers.

## 1 Introduction

Undernutrition and food insecurity remain major public health challenges in most developing nations. Globally, more than 815 million people are suffering from malnutrition or lack adequate access to food (1–3). In response to this issue, the United Nations declared 2016–2025 as the “Decade of Action on Nutrition” (4). Similarly, the Sustainable Development Goal (SDG) 2, “Zero Hunger,” aims to “ensure year-round access to safe, nutritious, and sufficient food for all” and “eliminate all forms of malnutrition” (5). Despite these global commitments, dietary quality continues to be inadequate in several low- and middle-income countries.

In rural areas, diets are often dominated by staple foods with limited inclusion of nutrient-rich foods, resulting in low dietary diversity and an increased risk of micronutrient deficiencies (6, 7). It is very challenging to achieve rural dietary diversity even though its importance for nutrient intake is well acknowledged because of the low literacy levels of the community, limited access to a variety of foods, and communal eating practices (8). These barriers highlight the need for simple, reliable measures that can capture the quality of individual diets in resource-poor environments.

Dietary diversity is defined as the number of different food groups consumed over a reference period, typically 24 h, and is not only a simple but also a very good indicator of diet quality and food access (9). When measured at the individual level, it reflects both the adequacy and the quality of dietary intake. Several studies have reported that dietary diversity scores (DDS) are reliable proxies for overall diet quality and household food security (10). Studies have also reported that dietary diversity is positively associated with nutritional status, including women’s nutritional status in rural Burkina Faso (11) and children’s nutrition in other low-income areas (12–14). Conversely, low dietary diversity is common among poor populations in developing countries, where diets are heavily reliant on starchy staples, and the intake of animal products, fruits, and vegetables is either restricted or dependent on the season (15). Recent national evidence further shows that India is experiencing a growing double burden of malnutrition, in which micronutrient deficiencies and undernutrition coexist with rising levels of overweight and obesity, partly linked to dietary diversity patterns and food fortification gaps (16). These issues are quite serious for rural labourers who need nutrient-rich foods to sustain physically demanding work, in addition to maintaining good health. Their food consumption pattern directly affects their health, productivity, and quality of life. Despite this, their diets often remain inadequate due to socio-demographic characteristics, cultural and historical food consumption patterns, the diversity of local farming systems, household income levels, and the availability of basic services such as clean water and healthcare (17, 18). Although several studies have explored dietary diversity among various populations in India, evidence focused specifically on rural labourers in South India remains limited. A high proportion of these labourers depend on agriculture for livelihood; income fluctuations, reliance on public food distribution systems, and region-specific constraints in accessing diversified foods further challenge adequate dietary intake. Addressing this gap is critical, as they face unique challenges and vulnerabilities in their work and living conditions. Keeping all these circumstances in mind, the present study aimed to assess dietary diversity among rural labourers in South India and identify the factors influencing it. In addition, the study further examined the relationship between dietary diversity and nutritional status, thereby providing evidence to inform nutrition interventions for this nutritionally vulnerable population.

*H_0_:* Dietary diversity and nutritional status of rural labourers are not related.

*H_1_:* Dietary diversity and nutritional status of rural labourers are related.

## 2 Materials and methods

### 2.1 Study area, period, and design

A community-based cross-sectional study was conducted from September 2023 to March 2024 in two South Indian states, i.e., Andhra Pradesh and Telangana, which were purposively selected due to their high worker population ratio (WPR) among rural labourers aged 15–59 years, as reported in the Periodic Labour Force Survey (20).

Andhra Pradesh, which has a total geographical area of 162,970 km^2^, was reorganized into 26 districts in 2022 and still largely relies on agriculture. The state’s major kharif crops are paddy, maize, millets, and pulses (19). Telangana, covering an area of 112,077 km^2^, cultivates approximately 27 major crops, including rice, maize, cotton, pulses, chillies, and sugarcane. The two states have three agricultural periods: harvest (October–December), post-harvest (January–April), and pre-harvest (May–September).

Rural labourers in these regions are primarily engaged in agricultural wage employment, performing heavy manual work informally and under insecure conditions. Although their contribution to agriculture is crucial, they often experience unemployment during off-seasons, earn meagre wages and have limited access to diversified foods. Therefore, they are very susceptible to food insecurity and nutritional stress, especially in the period before harvest when food is scarce. Their food intake consists mostly of cereals, with very low consumption of pulses, fruits, vegetables, and animal-source foods, resulting in micronutrient deficiencies and widespread undernutrition.

### 2.2 Sampling procedure

A multistage random sampling design was used. From each state, two districts were randomly selected, namely, YSR Kadapa and Annamayya in Andhra Pradesh, and Mahabubnagar and Khammam in Telangana. From each district, two blocks were selected, followed by two villages from each block. Within each village, 20 respondents (10 men and 10 women) were randomly selected from household lists maintained by the village panchayat offices, yielding a total sample of 320 respondents across the 4 districts.

The sample size was calculated using Cochran’s formula (21) for determining the sample size in large populations, with a 95% confidence level and a 5% margin of error:


$$n_{0}=\frac{Z^{2}p\left(1-p\right)}{e^{2}}$$

where:

*n*_0_ = required sample size; *Z* = 1.96 (for 95% confidence level); *p* = estimated proportion of the attribute in the population (0.5, assuming maximum variability); e = margin of error (0.05).

Thus, the minimum required sample size was 384 respondents. Considering the practical considerations related to field operations, finite population size, and potential non-response rate, the final sample size was adjusted to 320 respondents.

The study population comprised rural labourers residing in rural areas of Andhra Pradesh and Telangana, casually employed in agricultural and/or non-agricultural occupations, and paid wages in cash or kind based on daily or periodic contracts (excluding exchange labour). Only rural labourers with men aged 15–54 years and women aged 15–49 years (reproductive age excluding pregnant and lactating women), consistent with the (22) and the World Health Organization (WHO) guidelines, and those who were actively engaged in farming operations and household activities were included in the study after obtaining their informed consent. All respondents under 18 years of age provided verbal assent, and consent was obtained from a household head, as per the approved ethical guidelines.

### 2.3 Study variables

#### 2.3.1 Dietary diversity

Standard methods were used to conduct the dietary assessment, which included the 24-h dietary recall and the individual dietary diversity score (IDDS). Dietary diversity was a qualitative measure of food consumption and served as a proxy for food access and nutrient adequacy. The IDDS was calculated based on the number of food groups that an individual consumed during the 24 h prior to the survey, regardless of the quantities consumed (23). Non-typical days, such as festivals or celebrations, were not taken into account so that the dietary patterns represented usual intake (11).

The dietary diversity score was calculated based on the consumption of 10 food groups: (1) cereals and millets, (2) pulses and beans, (3) green leafy vegetables, (4) other vegetables, (5) roots and tubers, (6) fruits, (7) nuts, (8) milk and milk products, (9) fats and oils, and (10) meat and animal products (24). Each food group consumed was scored as 1, while a food group not consumed was scored as 0. The sum of all scores yielded the individual dietary diversity score (IDDS). The dietary diversity score (DDS) was categorized for analysis as low, medium, and high dietary diversity. A score of less than or equal to three food groups was classified as low dietary diversity (LDD), indicative of food insecurity; four to five food groups was considered medium dietary diversity (MDD), while a score of six or more food groups was classified as high dietary diversity (HDD), indicating food security (6).

#### 2.3.2 Anthropometric indices

Physical assessment of rural labourers was performed using anthropometric indices such as the body mass index (BMI), mid-upper arm circumference (MUAC), and calf circumference (CC).

The body mass index was derived by measuring the height and weight of the rural labourers using the following formula:


$$\mathrm{BMI}\;\left(\mathrm{kg}/m^{2}\right)=\frac{\mathrm{Weight}\;\left(\mathrm{kg}\right)}{{\mathrm{Height}}^{2}\;\left(m\right)}$$

Height was measured to the nearest 0.1 cm using a stadiometer with participants standing barefoot, and weight was measured to the nearest 0.1 kg using a calibrated digital scale with rural labourers in light clothing. Rural labourers were classified according to Garrow (25) as follows:

Classification of the BMI (25)

**Table:** 

BMI range	Presumptive diagnosis
<16.0	CED Grade III (severe)
16.0–17.0	CED Grade II (moderate)
17.0–18.5	CED Grade I (mild)
18.5–20.0	Low weight normal
20.0–25.0	Normal
25.0–30.0	Obese Grade I
>30	Obese Grade II

MUAC is a reliable screening indicator for protein–energy malnutrition, while CC serves as a proxy for muscle mass and functional reserves, especially in populations engaged in physical labour (26, 27). Measurements were taken using a non-stretchable measuring tape.

MUAC was measured at the midway point between the olecranon process of the ulna and the acromion process of the scapula. CC was measured as the maximum horizontal distance around the left calf as the rural labourer stood upright. MUAC and CC were analyzed by sex and divided into quantitative tertiles, as shown below.

Classification of MUAC (28)

**Table:** 

MUAC tertiles	For men	For women
I (undernutrition)	<22.9 cm	<22.8 cm
II (normal)	22.9–25.6 cm	22.8–25.4 cm
III (obese)	≥25.7 cm	≥25.5 cm

Classification of CC (29)

**Table:** 

CC tertiles	For men	For women
I (undernutrition)	<26.0 cm	<25.0 cm
II (normal)	26.0–29.9 cm	25.0–28.3 cm
III (obese)	≥30.0 cm	≥ 28.4 cm

### 2.4 Data collection tools and quality control

Data were collected through face-to-face interviews using a structured interview schedule. The schedule was pretested and underwent both content validation by experts from the Division of Agricultural Extension, ICAR–Indian Agricultural Research Institute, New Delhi, and face validation to ensure clarity and relevance of the items. Reliability was assessed through the pretest, and necessary modifications were made before final administration. The schedule consisted of concise, structured questions designed for easy comprehension. Primary data were gathered personally by the researcher, who also asked rural labourers to freely recall the food items and beverages they had consumed in the preceding 24 h, whether at home or outside. To increase the recall precision, the interviews were conducted in two stages: spontaneous recall and then probing questions to minimize the omissions.

Prior to data collection, the researcher spent a few days visiting each of the villages to establish rapport with the rural labourers through the help of the local extension staff, such as the village agriculture assistants, village horticulture assistants, and community leaders. Rural labourers were clearly informed that the participation was voluntary, and their data were used for academic purposes only.

A pilot study was conducted using 5% of the total sample from the area, which was excluded from the sample, resulting in very minor changes in the questions regarding their wording and clarity. The questionnaire was first written in English, translated into the regional language of the area (Telugu), and then checked by language experts to ensure consistency by translating it back into English. Data collection forms were checked for completeness and accuracy regularly before and during data entry.

### 2.5 Data processing and analysis

Data were entered into Microsoft Excel and later exported to R Studio (version 4.2.2) for statistical analysis. Variables were recoded as required. Descriptive statistics (frequencies, percentages, means, and standard deviations) were used to summarize the data. To determine the association between dietary diversity and nutritional status of rural labourers, Pearson’s chi-squared test was applied. Statistical significance was set at a *p*-value of <0.05. Finally, the results are presented in the form of text, tables, and figures for clarity and interpretation.

## 3 Results

### 3.1 Socio-demographic characteristics of rural labourers

In Andhra Pradesh, the mean age of rural labourers was 32 years, with an equal gender distribution (50% of men and 50% of women). The majority of rural labourers (85%) were married. The educational level was very low, with 35% being illiterate, followed by 30% completing primary education, 24.38% attending middle school, and only 10.63% having secondary education. The majority (80%) of rural labourers lived in nuclear families, while 20% belonged to joint families. The average family size was considered medium, consisting of four members. The majority (96.25%) of the rural labourers were engaged in agriculture as their main occupation, while a small proportion (3.75%) worked in non-agricultural labour. Their average monthly income was approximately ₹17,034.38, and their monthly per capita food expenditure was approximately ₹7,092.53. Nearly all the labourers (96.88%) were non-vegetarians, and only 3.13% were vegetarians.

In Telangana, the mean age of rural labourers was 34 years, with an equal gender distribution (50% of men and 50% of women). Approximately 77.5% were married. Educational levels were comparatively lower than in Andhra Pradesh, with 51.25% being illiterate, followed by 36.25% having primary education and middle school (12.5%). Nuclear families were common, with approximately 66.88%, followed by joint families (33.13%), with an average family size of four members. The majority (95.63%) of rural labourers were engaged primarily in agriculture, while 4.38% were engaged in non-agricultural occupations. The average monthly income was ₹8,625, and their monthly per capita food expenditure was approximately ₹6,693.45. Almost all rural labourers (98.13%) were non-vegetarians, and only 1.88% were vegetarians (Table 1).

**Table 1:** Socio-demographic characteristics of rural labourers in Andhra Pradesh and Telangana (*n* = 320).

Variables	Measurement	Andhra Pradesh (*n* = 160)		Telangana (*n* = 160)		*p*-value
		*f* (%)	Mean (SD)	*f* (%)	Mean (SD)	
Age (No. of years)	Chronological age (in years)	160 (100)	32.23 (8.90)	160 (100)	33.99 (8.78)	0.076
Sex	Male	80 (50)	–	80 (50)	–	–
	Female	80 (50)	–	80 (50)	–	
Marital status	Married	136 (85)	–	124 (77.5)	–	0.115
	Unmarried	24 (15)	–	36 (22.5)	–	
Education	Illiterate	56 (35.00)	–	82 (51.25)	–	0.0048
	Primary	48 (30.00)	–	58 (36.25)	–	
	Middle	39 (24.38)	–	20 (12.50)	–	
	Secondary	17 (10.63)	–	–	–	
	Higher secondary	–	–	–	–	
	College and above	–	–	–	–	
Family type	Nuclear family	128 (80)	–	107 (66.88)	–	0.011
	Joint family	32 (20)	–	53 (33.13)	–	
Family size	Total number of family members	–	4.24 (0.76)	–	4.00 (0.67)	0.0029
Occupation	Agriculture labour	154 (96.25)	–	153 (95.63)	–	–
	Non-agriculture labour	6 (3.75)	–	7 (4.38)	–	
Monthly income (Rs.)	Monthly income earned per month from agriculture and agriculture-related activities	–	17034.38 (5547.43)	–	8625.00 (2147.84)	<0.001
Monthly per capita expenditure on food (Rs.)	Total money spent on food items by the family members in the last 30 days.	–	7092.53 (440.69)	–	6693.45 (526.09)	<0.001
Dietary habits	Vegetarian	5 (3.13)	–	3 (1.88)	–	0.720
	Non-vegetarian	155 (96.88)	–	157 (98.13)	–	

State-wise comparisons were also conducted using independent t-tests for continuous variables and chi-squared tests for categorical variables. There was no significant difference in the mean age of rural labourers between Andhra Pradesh and Telangana (*p* = 0.076). Gender distribution was the same across both states, with equal proportions of men and women. The proportion of married rural labourers was slightly higher in Andhra Pradesh than in Telangana, but this difference was not statistically significant (*p* = 0.115). The educational level was significantly different between the two states (*p* = 0.0048), with a higher proportion of illiterate rural labourers in Telangana than in Andhra Pradesh. The family type also varied significantly (*p* = 0.011), with nuclear families being more prevalent in Andhra Pradesh and joint families being more common in Telangana. The average family size was slightly higher in Andhra Pradesh than in Telangana, and this difference was statistically significant (*p* = 0.0029). The majority of rural labourers in both states were engaged in agricultural labour, and no significant difference was observed in occupational distribution. However, monthly income (*p* < 0.001) and monthly per capita food expenditure (p < 0.001) were also significantly different. Dietary habits did not show a significant difference (*p* = 0.720), as nearly all rural labourers in both states reported being non-vegetarian (Table 1).

### 3.2 Physiological characteristics of rural labourers

The mean age of rural labourers was 32.23 ± 8.90 years in Andhra Pradesh and 33.99 ± 8.78 years in Telangana. The mean height was slightly higher in Telangana (155.00 ± 0.05 cm) than in Andhra Pradesh (154.00 ± 0.05 cm), whereas the mean weight (46.80 ± 7.08 kg vs. 45.86 ± 6.72 kg) and the BMI (19.77 ± 3.46 vs. 19.27 ± 3.22 kg/m^2^) were marginally higher in Andhra Pradesh. Measurements of mid-upper arm circumference and calf circumference were nearly identical across both states, indicating similar body composition patterns among labourers.

According to the NFHS-5 (2019–2021), 16.2% of rural women and 17.2% of rural men in Andhra Pradesh and 21.6% of rural women and 16.8% of rural men in Telangana were undernourished (BMI < 18.5 kg/m^2^). These findings are consistent with the results of the present study, confirming the persistent burden of chronic energy deficiency among rural labourers in both states (Table 2).

**Table 2:** Physiological characteristics among rural labourers (*n* = 320).

Physiological characteristics	Andhra Pradesh (*n* = 160)	Telangana (*n* = 160)
	Mean ± SD	Mean ± SD
Age (years)	32.23 ± 8.90	33.99 ± 8.78
Weight (kg)	46.80 ± 7.08	45.86 ± 6.72
Height (cm)	154.00 ± 0.05	155.00 ± 0.05
BMI (kg/m^2^)	19.77 ± 3.46	19.27 ± 3.22
MUAC (cm)	23.77 ± 2.05	23.71 ± 1.89
CC (cm)	26.71 ± 2.26	26.68 ± 2.14

### 3.3 Dietary diversity of rural labourers

The mean dietary diversity score (DDS) of rural labourers in Andhra Pradesh (4.63 ± 0.96) was slightly higher than that of Telangana (4.58 ± 1.07), indicating broadly similar dietary patterns across both states, with the majority of rural labourers consuming four to five food groups on the preceding day (Table 3).

**Table 3:** Categorisation of rural labourers based on dietary diversity in Andhra Pradesh and Telangana.

Dietary diversity	Andhra Pradesh (*n* = 160)				Telangana (*n* = 160)			
	Men (*n* = 80)		Women (*n* = 80)		Men (*n* = 80)		Women (*n* = 80)	
	*f*	%	*f*	%	*f*	%	*f*	%
Low dietary diversity (≤3 food groups)	12	15.00	16	20.00	12	15.00	24	30.00
Medium dietary diversity (4 and 5 food groups)	51	63.75	63	78.75	55	68.75	42	52.50
High dietary diversity (≥6 food groups)	17	21.25	1	1.25	13	16.25	14	17.50
Pearson’s chi-squared test	*χ*^2^ = 23.56, d*f* = 6, *p*-value = 0.0006							

**Figure 1:**
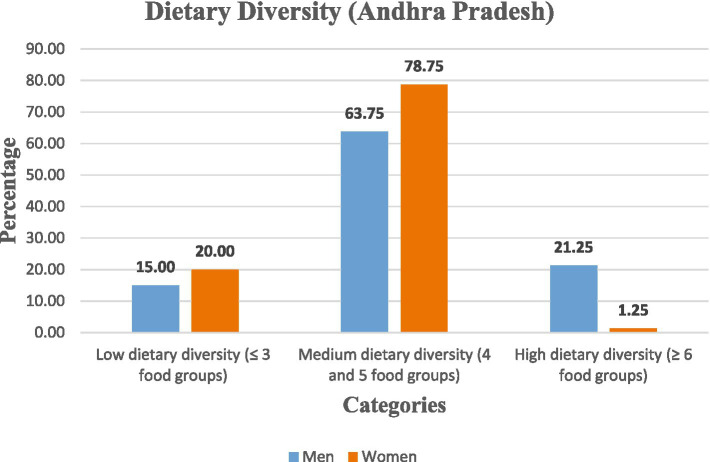
Categorisation of rural labourers based on dietary diversity in Andhra Pradesh.

**Figure 2:**
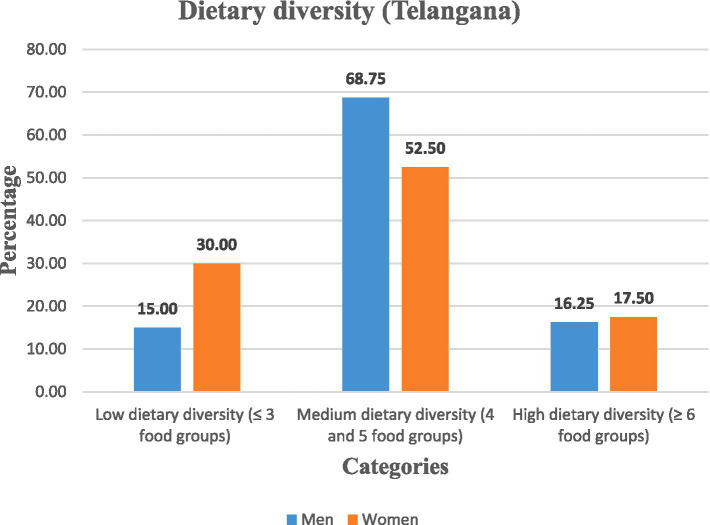
Categorisation of rural labourers based on dietary diversity in Telangana.

Gender-wise analysis was conducted within the states. In Andhra Pradesh, the majority of women (78.75%) and men (63.75%) fell within the medium dietary diversity category (4–5 food groups). However, the majority of women (20.00%) had lower dietary diversity than men (15.00%), while men had a relatively better dietary diversity than women, with a greater proportion (21.25%) attaining high diversity (≥6 food groups) compared to only 1.25% of women. In Telangana, although the overall pattern was similar, gender disparities were narrower. The majority of men (68.75%) and women (52.50%) had medium dietary diversity, but women had relatively better representation in the high dietary diversity group (17.50%) compared to Andhra Pradesh, suggesting improved dietary access (Figures 1, 2).

The chi-squared test revealed a significant association between gender, state, and dietary diversity categories (*χ*^2^ = 23.56, d*f* = 6, *p* = 0.0006), indicating that these differences are statistically valid (Table 3).

Therefore, the findings revealed that dietary diversity was medium among rural labourers, with gender disparities more pronounced in Andhra Pradesh, where women were disproportionately concentrated in low dietary diversity categories.

### 3.4 Nutritional status of rural labourers

The nutritional status of rural labourers was measured using different anthropometric indicators, namely, body mass index (BMI), mid-upper arm circumference (MUAC), and calf circumference (CC) (Tables 4–6).

**Table 4:** Prevalence (%) of chronic energy deficiency (CED) among rural labourers in Andhra Pradesh and Telangana (*n* = 320).

BMI range	BMI grades	Andhra Pradesh (*n* = 160)				Telangana (*n* = 160)			
		Men (*n* = 80)		Women (*n* = 80)		Men (*n* = 80)		Women (*n* = 80)	
		*f*	%	*f*	%	*f*	%	*f*	%
<16.0	CED Grade III (severe)	9	11.25	10	12.50	8	10.00	11	13.75
16.0–17.0	CED Grade II (moderate)	7	8.75	9	11.25	15	18.75	19	23.75
17.0–18.5	CED Grade I (mild)	19	23.75	21	26.25	12	15.00	13	16.25
18.5–20.0	Low weight normal	20	25.00	19	23.75	21	26.25	20	25.00
20.0–25.0	Normal	18	22.50	16	20.00	17	21.25	15	18.75
25.0–30.0	Obese Grade I	4	5.00	3	3.75	4	5.00	1	1.25
>30	Obese Grade II	3	3.75	2	2.50	3	3.75	1	1.25

**Table 5:** Distribution of respondents based on mid-upper arm circumference (MUAC) among rural labourers in Andhra Pradesh and Telangana.

MUAC tertiles	For men	For women	Andhra Pradesh (*n* = 160)				Telangana (*n* = 160)			
			Men (*n* = 80)		Women (*n* = 80)		Men (*n* = 80)		Women (*n* = 80)	
			*f*	%	*f*	%	*f*	%	*f*	%
I (undernutrition)	<22.9 cm	<22.8 cm	40	50.00	49	61.25	38	47.50	48	60.00
II (normal)	22.9–25.6 cm	22.8–25.4 cm	33	41.25	27	33.75	35	43.75	30	37.50
III (obese)	≥25.7 cm	≥ 25.5	7	8.75	4	5.00	7	8.75	2	2.50

**Table 6:** Distribution of respondents based on calf circumference (CC) among rural labourers in Andhra Pradesh and Telangana.

CC tertiles	For men	For women	Andhra Pradesh (*n* = 160)				Telangana (*n* = 160)			
			Men (*n* = 80)		Women (*n* = 80)		Men (*n* = 80)		Women (*n* = 80)	
			*f*	%	*f*	%	*f*	%	*f*	%
I (undernutrition)	<26.0 cm	<25.0 cm	46	57.50	40	50.00	39	48.75	35	43.75
II (normal)	26.0–29.9 cm	25.0–28.3 cm	29	36.25	33	41.25	34	42.50	36	45.00
III (obese)	≥30.0 cm	≥28.4 cm	5	6.25	7	8.75	7	8.75	9	11.25

#### 3.4.1 Body mass index

**Figure 3:**
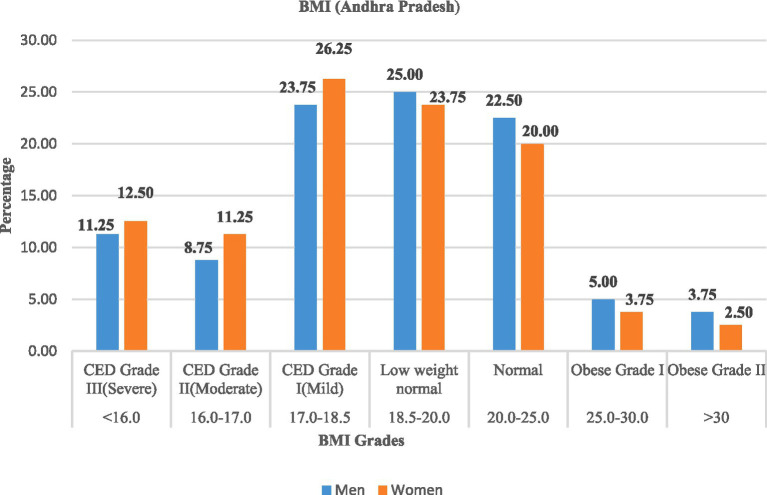
Prevalence (%) of chronic energy deficiency (CED) among rural labourers in Andhra Pradesh.

**Figure 4:**
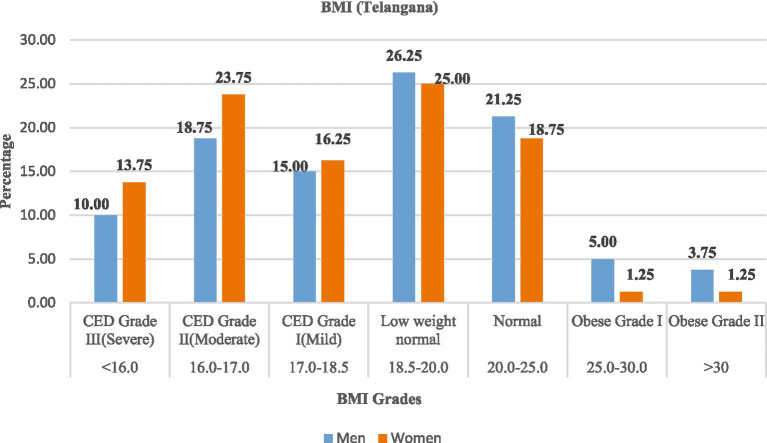
Prevalence (%) of chronic energy deficiency (CED) among rural labourers in Telangana.

A high prevalence of chronic energy deficiency (CED) was observed in both the states, as shown in Table 4, Figures 3, 4. In Andhra Pradesh, the majority of women (50.00%) and men (43.75%) were affected by CED, with Grade I (mild) being the most prevalent. Only approximately one-fifth of the rural labourers were classified as normal weight (20.0% of women and 22.50% of men), while 23.75% of women and 25.00% of men were classified as low-weight normal category, and very few (<6%) were obese. In Telangana, the prevalence of CED remained high (43.75% of men; 53.75% of women), with a slightly lower share of normal-weight rural labourers. Severe CED (Grade III) was more frequent among women in both states.

These findings highlighted the substantial burden of undernutrition, especially in the case of women, among the rural labourers, while the occurrence of obesity is almost negligible.

#### 3.4.2 Mid-upper arm circumference

**Figure 5:**
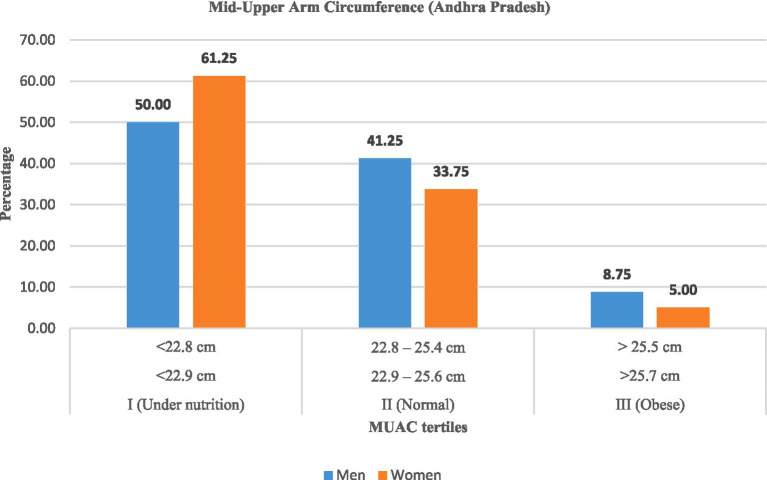
Mid-upper arm circumference (MUAC) among rural labourers in Andhra Pradesh.

**Figure 6:**
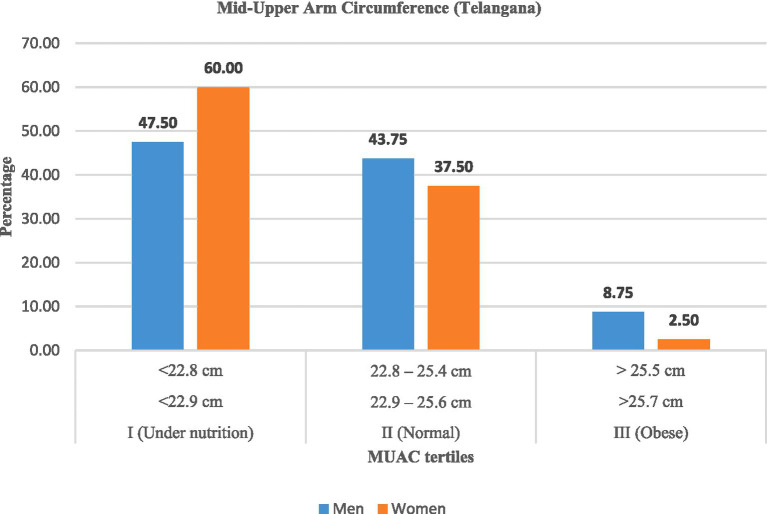
Mid-upper arm circumference (MUAC) among rural labourers in Telangana.

Mid-upper arm circumference (MUAC) measurements (Table 5; Figures 5, 6) confirmed widespread undernutrition. In Andhra Pradesh, the majority of women (61.25%) and men (50.00%) were undernourished (MUAC measurement was <22.9 cm for men and <22.8 cm for women), compared with 60.00% of women and 47.50% of men in Telangana. The proportion of normal MUAC ranged between 33 and 44%, while obesity was rare (<9%).

These findings indicated that women labourers were more likely to be undernourished than men across both states, although interstate variation was minimal.

#### 3.4.3 Calf circumference

**Figure 7:**
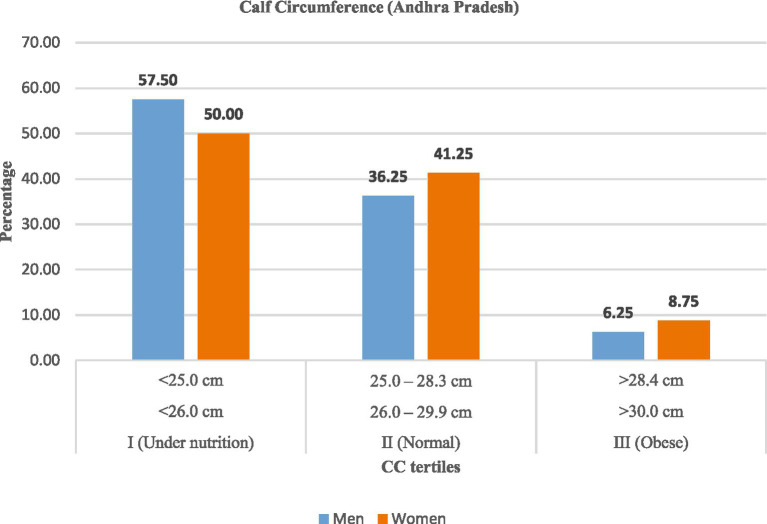
Calf circumference (CC) among rural labourers in Andhra Pradesh.

**Figure 8:**
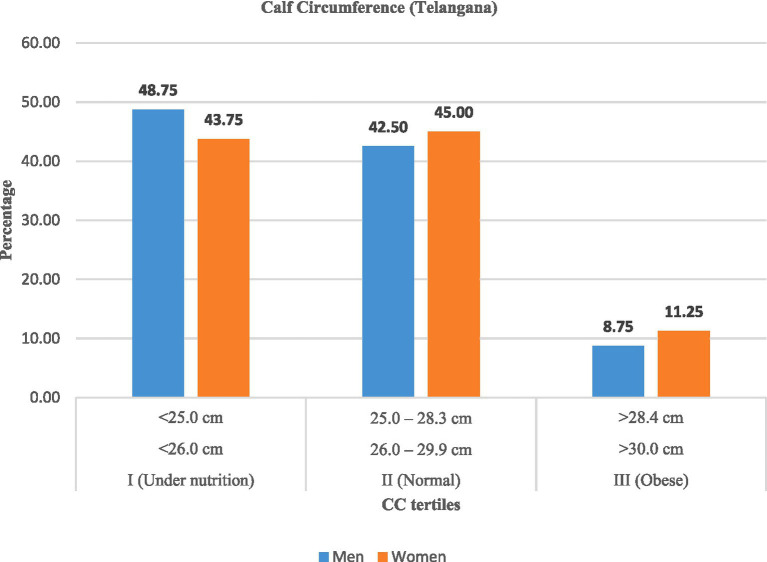
Calf circumference (CC) among rural labourers in Telangana.

Similarly, calf circumference (CC) data (Table 6; Figures 7, 8) revealed high levels of undernutrition. In Andhra Pradesh, the majority (57.50%) of men and (50.00%) of women were undernourished, while 36–41% were normal. In Telangana, undernutrition was slightly lower (48.75% of men; 43.75% of women) with higher proportions in the normal category (42 to 45%). Obesity remained uncommon (<11%).

Overall, men were more likely to exhibit lower calf circumference, reflecting reduced muscle mass and nutritional reserves, whereas women in Telangana observed relatively better muscle status.

### 3.5 Relationship between dietary diversity and nutritional status of rural labourers

The association between dietary diversity and nutritional status, assessed using anthropometric indicators (BMI, MUAC, and CC), was examined using Pearson’s chi-squared test in R Studio (version 4.2.2). The results are presented in Tables 7–9.

**Table 7:** Dietary diversity on the body mass index (BMI) of rural labourers in Andhra Pradesh and Telangana.

BMI grades	Andhra Pradesh (*n* = 160)			Telangana (*n* = 160)		
	Lowest dietary diversity (≤3 food groups)	Medium dietary diversity (4 and 5 food groups)	High dietary diversity (≥6 food groups)	Lowest dietary diversity (≤3 food groups)	Medium dietary diversity (4 and 5 food groups)	High dietary diversity (≥6 food groups)
CED Grade III (severe)	17	2	0	19	0	0
CED Grade II (moderate)	11	5	0	17	4	0
CED Grade I (mild)	0	40	1	0	32	0
Low weight normal	0	37	0	0	40	0
Normal	0	26	9	0	21	18
Obese Grade I	0	3	4	0	0	5
Obese Grade II	0	1	4	0	0	4
Pearson’s chi-squared test	*χ*^2^ = 178.06, d*f* = 12, *p*-value <2.2e−16			*χ*^2^ = 226.84, d*f* = 12, *p*-value <2.2e−16		

CED, chronic energy deficiency.

**Table 8:** Dietary diversity on mid-upper arm circumference (MUAC) of rural labourers in Andhra Pradesh and Telangana.

MUAC tertiles	Andhra Pradesh (*n* = 160)			Telangana (*n* = 160)		
	Lowest dietary diversity (≤3 food groups)	Medium dietary diversity (4 and 5 food groups)	High dietary diversity (≥6 food groups)	Lowest dietary diversity (≤3 food groups)	Medium dietary diversity (4 and 5 food groups)	High dietary diversity (≥6 food groups)
I (undernutrition)	28	61	1	36	50	0
II (normal)	0	50	9	0	47	18
III (obese)	0	3	8	0	0	9
Pearson’s chi-squared test	*χ*^2^ = 72.443, d*f* = 4, *p*-value = 6.917e−15			*χ*^2^ = 93.856, d*f* = 4, *p*-value <2.2e−16		

**Table 9:** Dietary diversity on calf circumference (CC) of rural labourers in Andhra Pradesh and Telangana.

CC tertiles	Andhra Pradesh (*n* = 160)			Telangana (*n* = 160)		
	Lowest dietary diversity (≤3 food groups)	Medium dietary diversity (4 and 5 food groups)	High dietary diversity (≥6 food groups)	Lowest dietary diversity (≤3 food groups)	Medium dietary diversity (4 and 5 food groups)	High dietary diversity (≥6 food groups)
I (undernutrition)	28	55	1	35	36	3
II (normal)	0	53	11	1	55	14
III (obese)	0	6	6	0	6	10
Pearson’s chi-squared test	*χ*^2^ = 53.266, d*f* = 4, *p*-value = 7.497e−11			*χ*^2^ = 71.868, d*f* = 4, *p*-value = 9.15e−15		

CC, calf circumference; DD, dietary diversity.

#### 3.5.1 Effect of dietary diversity on body mass index

**Figure 9:**
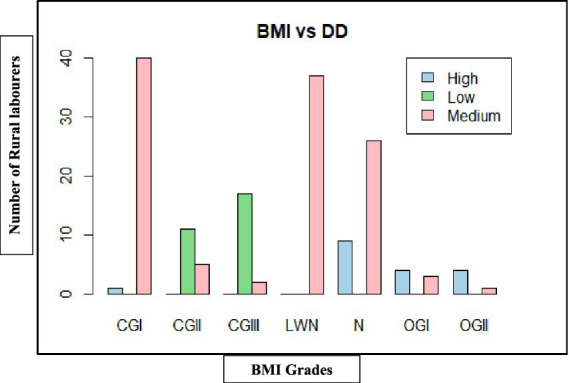
Dietary diversity on body mass index (BMI) of rural labourers in Andhra Pradesh.

**Figure 10:**
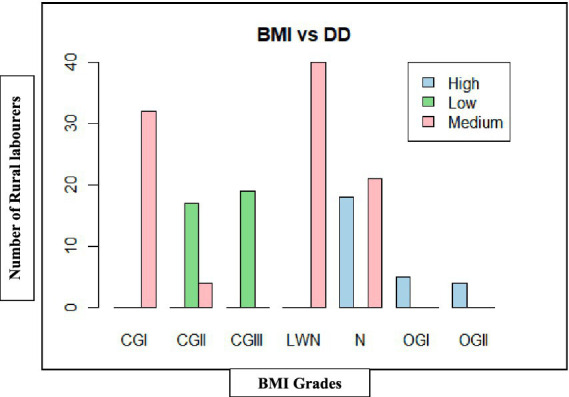
Dietary diversity on body mass index (BMI) of rural labourers in Telangana. BMI, body mass index; DD, dietary diversity; CGI, chronic energy deficiency Grade I (Mild); CGII, chronic energy deficiency Grade II (Moderate); CGIII, chronic energy deficiency Grade III (Severe); LWN, low weight normal; N, normal; OGI, Obese Grade I; OGII, Obese Grade II.

As shown in Table 7, Figures 9, 10, rural labourers with low dietary diversity (≤3 food groups) were exclusively undernourished, with most falling into chronic energy deficiency (CED) Grade II (moderate) or CED III (severe). Those with medium dietary diversity (4–5 food groups) had mixed outcomes, including mild CED and low-weight normal categories. Rural labourers with high dietary diversity (≥6 food groups) were predominantly normal or overweight.

The association between dietary diversity and BMI was highly significant in both states, Andhra Pradesh (*χ*^2^ = 178.06, d*f* = 12, *p* < 0.001) and Telangana (*χ*^2^ = 226.84, d*f* = 12, *p* < 0.001), confirming that improved dietary diversity was strongly linked to better BMI outcomes.

#### 3.5.2 Effect of dietary diversity on mid-upper arm circumference

**Figure 11:**
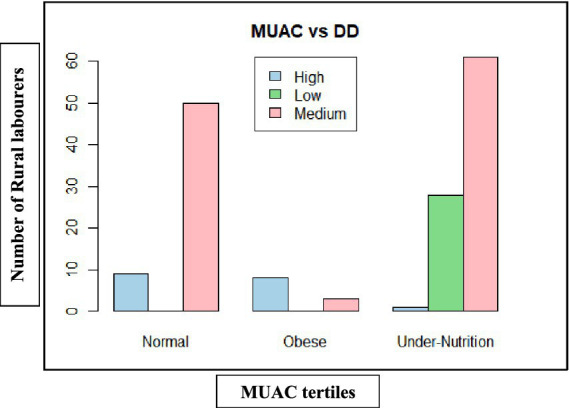
Dietary diversity on mid-upper arm circumference (MUAC) of rural labourers in Andhra Pradesh.

**Figure 12:**
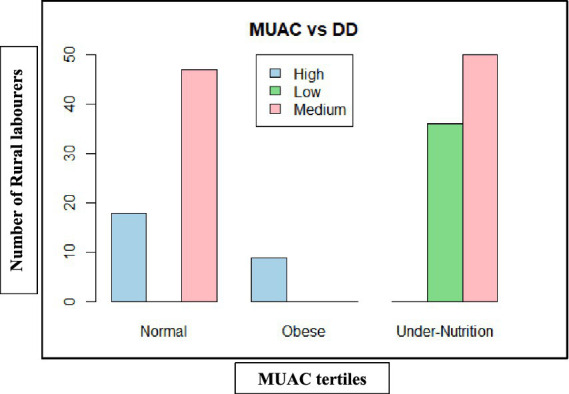
Dietary diversity on mid-upper arm circumference (MUAC) of rural labourers in Telangana. MUAC, mid-upper arm circumference; DD, dietary diversity.

Mid-upper arm circumference (MUAC)-based nutritional outcomes (Table 8, Figures 11, 12) also had a similar pattern. In both states, low dietary diversity corresponded entirely to undernutrition, while high dietary diversity aligned with normal or obese MUAC tertiles. The relationship was statistically significant in Andhra Pradesh (*χ*^2^ = 72.44, d*f* = 4, *p* < 0.001) and Telangana (*χ*^2^ = 93.85, d*f* = 4, *p* < 0.001), highlighting the consistent positive influence of dietary diversity on muscle and fat reserves.

#### 3.5.3 Effect of dietary diversity on calf circumference

**Figure 13:**
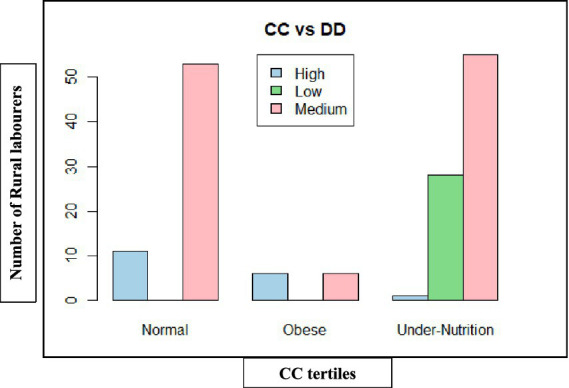
Dietary diversity on calf circumference (CC) of rural labourers in Andhra Pradesh.

**Figure 14:**
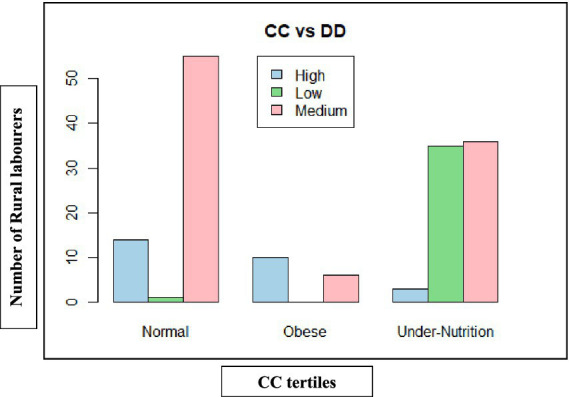
Dietary diversity on calf circumference (CC) of rural labourers in Telangana. CC, calf circumference; DD, dietary diversity.

Calf circumference-based classification (Table 9, Figures 13, 14) also reinforced these findings. In both states, low dietary diversity corresponded almost entirely to undernutrition, whereas high dietary diversity was associated with improved muscle mass and nutritional status. The association was statistically significant in Andhra Pradesh (*χ*^2^ = 53.27, d*f* = 4, *p* < 0.001) and Telangana (*χ*^2^ = 71.87, d*f* = 4, *p* < 0.001).

### 3.6 Factors influencing dietary diversity

Multiple linear regression analysis was conducted to examine the effect of socio-demographic and physiological factors on dietary diversity among rural labourers (Table 10). The results revealed a strong model fit (*R*^2^ = 0.77; adjusted *R*^2^ = 0.76), and the overall regression was statistically significant (*p* < 0.001), indicating that the selected variables explained 76% of the variation in dietary diversity among rural labourers.

**Table 10:** Multiple linear regression analysis of socio-demographic characteristics associated with the dietary diversity of rural labourers.

Variables	*β* (unstandardized coefficients)		*t*-value	*p*-value
	Estimate	Standard error		
Intercept	−3.448	0.960	−3.593	0.00038***
Age	0.025	0.010	2.397	0.0171*
Education	0.130	0.023	5.721	<0.001***
Family size	−0.090	0.051	−1.763	0.0799.
Monthly income	0.0000104	0.0000063	1.649	0.1001
Monthly per capita expenditure on food (Rs.)	0.0000215	0.0000732	0.294	0.7691
Body mass index	0.00127	0.0234	5.374	<0.001***
Mid-upper arm circumference	0.1197	0.0300	3.994	<0.001***
Calf circumference	0.1295	0.0245	5.276	<0.001***
**Model summary**				
Residual standard error	0.4949	d*f*	310	***p*-value**
***R*** ^**2**^	0.77	**Adjusted *R*** ^**2**^	0.76	<0.001
*F*-statistic	113.6***	d*f*	9 and 310	

BMI, body mass index; MUAC, mid-upper arm circumference; CC, calf circumference; MPE_food, monthly per capita expenditure on food.

‘***’0.001, ‘**’0.01, ‘*’0.05, ‘.’0.1.

Among the predictors, education was found to be the strongest positive predictor of dietary diversity (*β* = 0.130, *p* < 0.001), which indicates that the rural labourers with higher levels of education had better dietary diversity. Age was also a contributor to the dietary diversity, but it showed only a slight positive effect that was statistically significant (*β* = 0.025, *p* = 0.017). In terms of anthropometric indices, the BMI (*p* < 0.001), mid-upper arm circumference (*p* < 0.001), and calf circumference (*p* < 0.001) were strong positive predictors of dietary diversity, thus suggesting that improved nutritional status was associated with higher dietary diversity. However, family size (*p* = 0.080), monthly income (*p* = 0.100), and monthly per capita food expenditure (*p* = 0.769) were not significant predictors of dietary diversity.

These findings confirmed that socio-demographic characteristics and dietary diversity are significantly related among rural labourers.

## 4 Discussion

The present study highlights the critical role of dietary diversity in shaping the nutritional status of rural labourers in Andhra Pradesh and Telangana. Although the BMI is one of the most widely used anthropometric indices for assessing undernutrition, it still possesses various limitations, which are acknowledged widely. The measurement does not consider the components of body weight, such as muscle mass, fat mass, and bone mass; therefore, rural labourers with a higher bone density or greater muscle mass may be misclassified as having a better nutritional status. These limitations are particularly relevant for rural labourers engaged in strenuous physical work, as the BMI may not be able to capture the differences in body composition that exist in this group. For this reason, the present study incorporated not only BMI but also the measurements of MUAC and calf circumference, which offer more precise estimates of muscle mass, peripheral fat stores, and functional reserves, thus reinforcing the accuracy of nutritional assessment.

The mean dietary diversity scores (DDS) among rural labourers were predominantly in the medium category, indicating that the rural labourers consumed on average approximately four to five food groups on the reference day. The predominance of medium DDS across both states indicates that rural labourers had access to some variety in their diets, with the limited inclusion of nutrient-rich food groups such as pulses, vegetables, fruits, dairy, and animal-source foods. Thus, this raises serious concerns about micronutrient adequacy and overall diet quality. The findings were in line with the report of Food and Agriculture Organization of United Nations (FAO) (30).

In the case of dietary diversity assessment, more men were found in the high dietary diversity category than women. This trend was very much pronounced in the state of Andhra Pradesh, where only 1.25% of women had high dietary diversity compared to 21.25% of men, indicating an intra-household gender imbalance in food access and allocation. Harris Fry et al. (31) reported similar findings in South Asia, where women’s greater vulnerability to undernutrition is well-documented and is primarily attributed to inequitable intra-household food distribution, cultural norms that prioritize men’s food consumption, limited decision-making power, heavier unpaid workload, and reproductive health stresses. These social and biological factors contribute more to undernutrition than higher energy requirements alone. These factors explain why women are disproportionately affected by undernutrition despite performing substantial physical work. However, in Telangana, the results revealed a relatively narrower gender gap in the high dietary diversity category, with nearly similar proportions of men and women attaining high dietary diversity. Nevertheless, a substantial proportion of women in Telangana are still clustered in the low dietary diversity category, indicating greater vulnerability to dietary inadequacies. This suggests that while some women in Telangana could access a diverse diet, others remained vulnerable, highlighting inequalities within and between households. These findings are consistent with evidence that women’s access to food is limited by socio-cultural norms, economic marginalization, and structural barriers, which collectively worsen their nutritional outcomes (32, 33).

The nutritional assessment revealed a high prevalence of chronic energy deficiency (CED) among rural labourers, with BMI values below 18.5 kg/m^2^. This undernutrition was particularly pronounced among women, which is consistent with the NFHS-5 (2019–21) data for rural areas of Andhra Pradesh and Telangana. The observed gender disparity can be attributed to factors such as inequitable food distribution, heavy physical workload, reproductive health issues, and limited healthcare access. The findings align with the results of Joshi et al. (34), who reported that seasonal fluctuations in agricultural employment, heavy physical workload, inadequate dietary intake, and increased vulnerability to nutritional deficiencies among women contribute to chronic undernutrition. The BMI distribution revealed a large proportion of rural labourers in the low-weight normal category (18.5–20.0 kg/m^2^), indicating that there are potential physiological vulnerabilities and an elevated risk of undernutrition during agricultural lean seasons. On the other hand, the presence of overweight and obesity in this population was rare, indicating that physically demanding nature of agricultural work and, therefore, possible dietary energy might be insufficient. This finding is consistent with the results of Subramanian et al. (35).

Mid-upper arm circumference and calf circumference were observed to have similar trends and are recognized as robust indicators of protein–energy status and muscle mass, especially in physically active populations, as they reflect the nutritional status and functional reserves that were reported by the World Health Organization (27), Bonnefoy et al. (36), and Rolland et al. (26). In this study, the results of MUAC data revealed a widespread prevalence of undernutrition, particularly among women, thus confirming the cumulative effects of dietary inadequacy, irregular meal frequency, and high energy expenditure associated with manual agricultural work. These findings were in line with the results of Chakraborty and Bose (37), who reported similar patterns among female agricultural labourers in rural West Bengal. Additionally, calf circumference data revealed poorer muscle mass among rural labourers in Andhra Pradesh compared to Telangana, implying potential differences in food access, dietary intake, physical workload, and access to nutrition interventions. Multiple contextual factors may contribute to these interstate differences. Variations exist in livelihood patterns, levels of mechanization in agricultural labour, and access to social welfare schemes such as the Public Distribution System (PDS), Integrated Child Development Services (ICDS), and the Mahatma Gandhi National Rural Employment Guarantee Scheme (MGNREGS). Variations in agricultural diversity and access to nutrient-dense food markets may also influence dietary preferences. Moreover, socio-economic conditions, such as education level, women’s empowerment, and the stability of household income, are different between the two states and likely contribute to discrepancies in the consumption of diverse diets and nutritive outcomes. Taking these anthropometric indicators together indicates that the women agricultural labourers are doubly vulnerable, as they always had poor nutritional indices. These findings are consistent with the results of Joshi et al. (38).

Pearson’s chi-squared test revealed a strong and significant association between dietary diversity and nutritional status. Rural labourers with low dietary diversity (i.e., ≤3 food groups) were consistently undernourished, indicating that a diet dominated by cereals fail to meet nutritional requirements. This finding is in line with the results of Arimond and Ruel (15) and Kennedy et al. (39). On the other hand, rural labourers with high dietary diversity (≥6 food groups) had normal or improved nutritional status, which is in line with the global findings that greater dietary diversity enhances nutrient adequacy and reduces undernutrition (40). However, the presence of overweight/obesity among rural labourers with high dietary diversity also points to the emerging double burden of malnutrition.

Higher dietary diversity is, in general, associated with improved micronutrient adequacy and reduced risk of undernutrition; however, it does not necessarily prevent overnutrition. Rural labourers with high dietary diversity may still consume energy-dense foods or eat in quantities that exceed their caloric requirements. When the calories consumed are higher than those expended, weight gain can occur, irrespective of the variety in the diet. This suggests that the small proportion of overweight rural labourers in the high DDS group may reflect a scenario where dietary variety coexists with excess calorie consumption. This pattern is increasingly observed in populations undergoing nutritional transition. Incorporating these findings reinforces the relevance of our results, particularly the coexistence of undernutrition and emerging overweight among rural labourers, where dietary improvement can coexist with increasing risks of overweight in rural populations. This finding is in line with the study by Popkin et al. (41) and Jindal et al. (16).

In the present study, state-level variations further highlight the complex relationship between dietary diversity and nutritional status. A state-level difference was observed in the medium dietary diversity category. In Andhra Pradesh, medium dietary diversity was associated with both undernutrition and normal/obese categories, suggesting a transitional dietary shift. Conversely, in Telangana, medium dietary diversity was closely related to undernutrition, implying that the nutritional benefits from medium dietary improvements were not limited. These differences may be due to various factors such as food availability, market access, agricultural production, and household-level food allocation practices across the two states. The findings are in line with the findings of Swindale and Bilinsky (42) and Kadiyala et al. (32).

Multiple linear regression analysis has revealed that the socio-demographic and nutritional factors together explained 76% (adjusted *R*^2^ = 0.76) of the variation in dietary diversity among rural labourers. Education emerged as a particularly strong determinant, suggesting that individuals with higher educational attainment are likely to be more knowledgeable about informed food choices and hence consume a greater variety of foods. Similar results have been reported by Arimond and Ruel (15), who, in contrast, argued that maternal and household education significantly influence dietary quality and nutrition within families. Anthropometric indicators such as body mass index, mid-upper arm circumference, and calf circumference were also positively associated with dietary diversity, implying that people in better nutritional health are likely to have more diverse diets. These results are consistent with the research studies by Rolland et al. (26) and the World Health Organization (27), who also highlight the role of muscle mass and nutritional status in determining overall dietary adequacy. On the other hand, family size had a negative relationship with the dietary diversity, reflecting the dilution of household resources when family size increases, a trend also observed in rural nutrition studies by Hoddinott and Yohannes (10). Variables such as age, income, and per capita food expenditure exhibited limited influence, possibly due to the uniformity of economic conditions among the respondents.

The regression analysis confirms that socio-demographic characteristics and anthropometric indicators significantly influence dietary diversity among rural labourers, emphasizing that higher education levels, improved nutritional health, and better socio-economic conditions collectively contribute to more diversified diets. Hence, the present study reinforces the critical role of socio-demographic factors in shaping food choices and overall nutritional status.

Finally, the study concludes that dietary diversity is a significant factor of nutritional status among rural labourers. The consistent association of high dietary diversity with improved BMI, MUAC, and CC underscores the multidimensional importance of diet quality in maintaining energy balance, muscle mass, and functional health. Persistent gender inequities and the emerging risks of overweight highlight the dual challenges of not only combating undernutrition but also preventing diet-related diseases associated with non-communicable diseases in rural India.

## 5 Conclusion

The present study revealed a high prevalence of undernutrition and low dietary diversity among rural labourers in Andhra Pradesh and Telangana. The findings clearly indicated that dietary diversity is a significant determinant of nutritional status, exerting a strong influence on anthropometric indicators. Among the rural labourers, low dietary diversity was generally observed, which highlights the need for nutrition education and the awareness of the importance of consuming various food groups and maintaining balanced diets, which can play a crucial role in reducing chronic energy deficiency and improving overall health. Women, particularly in Andhra Pradesh, emerged as the most nutritionally disadvantaged group, reflecting the persistence of structural and cultural inequities that limit their access to diverse and nutritious foods. Therefore, nutrition-sensitive interventions must go beyond food availability to address underlying determinants such as poverty, gender inequality, food affordability, and intra-household food distribution.

### 5.1 Policy implications

Policy measures should focus on:

- Promoting dietary diversification through improved access to pulses, fruits, vegetables, and animal-source foods.
- Strengthening women’s empowerment and their participation in decision-making about household food choices.
- Enhancing livelihood opportunities to ensure stable incomes and food security throughout the year and
- Integrating nutrition education along with rural development and agricultural extension programmes.

Additionally, community-based strategies such as nutrition and health awareness campaigns, promotion of nutrition gardens, and routine anthropometric monitoring can help policymakers in evaluating the effectiveness of the Public Distribution System (PDS) and designing targeted nutrition interventions to improve dietary intake and nutritional status among rural labourers.

#### 5.1.1 Limitations of the study

- The study has the following limitations: The study focused on rural labourers in Andhra Pradesh and Telangana, and therefore, the findings may not be generalizable to other regions or farming communities with different socio-economic and cultural contexts.
- Since the majority of the respondents did not manage farm records, their comments depended on their ability to remember facts, which could have influenced the impartiality of their responses and, consequently, the study’s findings.
- The study was limited by constraints related to time, financial resources, and conveyance facilities during the investigation.
- Some aspects of the study would benefit from a longitudinal approach, involving observation and data collection over time to provide a clear picture. However, this was not feasible in this study.

### 5.2 Future research

Future studies should evaluate the effectiveness of ongoing nutrition intervention programmes and policies in improving the nutritional status of rural labourers. Moreover, research will also be required to see the extent to which agricultural innovations such as crop diversification, biofortification, and value chain improvements can increase food security and dietary quality. Longitudinal and mixed-method studies considering the socio-economic, cultural, and behavioral dimensions would provide deeper insights into sustainable strategies for achieving nutritional resilience in rural populations.

## Data Availability

The raw data supporting the conclusions of this article will be made available by the authors, without undue reservation.
